# Effect of photobiomodulation on lower urinary tract dysfunction in rat cystitis model

**DOI:** 10.1371/journal.pone.0306527

**Published:** 2024-07-26

**Authors:** Naoya Ishibashi, Tomoyuki Uchiyama, Shinichi Tao

**Affiliations:** 1 Bio-Medical Engineering Group, Drug Discovery Laboratory, Teijin Institute for Bio-Medical Research, Teijin Pharma Ltd., Tokyo, Japan; 2 Department of Neurology, School of Medicine, International University of Health and Welfare, Chiba, Japan; 3 Department of Neurology, International University of Health and Welfare Shioya Hospital, Tochigi, Japan; 4 Department of Neurology, School of Medicine, Chiba University, Chiba, Japan; Massachusetts General Hospital, UNITED STATES

## Abstract

**Objective:**

Photobiomodulation selectively controls the activity of the sensory nervous system associated with A-delta and C fibers. Hypersensitivity involving the afferent A-delta and C fibers occurs in cystitis and decreases urinary function. This study aimed to investigate the effect of photobiomodulation on urinary storage dysfunction and voiding functions in cystitis model rats.

**Methods:**

We prepared the rat cystitis model. Under anesthesia, a cannula was connected to the bladder via a ventral incision. 0.3% acetic acid or saline was injected into the bladder. Continuous cystometry was performed, measuring bladder pressure and voiding urine volume with rats freely mobile. Laser irradiation was applied to the L6 lumbosacral intervertebral foramen using an 830 nm laser. Residual urine was extracted post-cystometry.

**Results:**

In the rat cystitis model groups, there was a significant decrease in the voiding interval and volume compared to the group receiving normal saline infusion. After sham or laser irradiation, only the group with laser irradiation showed a significant increase in voiding interval (217%, *p* = 0.0002) and voiding volume (192%, *p* = 0.0012) in the parameters of storage dysfunction. The basal pressure, intravesical pressure, and residual urine volume remained unchanged in all groups before and after irradiation.

**Conclusions:**

This study indicates that photobiomodulation may improve urine storage dysfunction without exacerbating voiding function in a rat model of cystitis. Thus, photobiomodulation may be a new treatment option for the hypersensitivity and detrusor overactivity caused by cystitis.

## Introduction

Photobiomodulation (PBM) or low-level laser therapy (LLLT) have been used in clinical practice for several decades to treat many painful conditions [1, 2]. Over 100 randomized, double-blind, placebo-controlled clinical trials (RCTs) have been published. Many RCTs have shown positive outcomes for osteoarthritis [3], tendinopathies [4], wounds [5], back pain [6], neck pain [7], muscle fatigue [8], peripheral nerve injuries [9], and stroke [10]. Basic supportive studies investigating the mechanisms, local tissues, and systemic effects have also been published. The anti-inflammatory effects, accelerated bone nodule formation, pain relief, and nerve repair induced by laser irradiation have been reported *in vitro* and *in vivo* [2]. Some *in vivo* studies have reported that laser irradiation can control peripheral sensory nervous system activity associated with A-delta and C fibers [11–17].

The sensory nervous system functions as an important trigger, playing a role not only in pain perception but also in bladder control. Symptoms associated with detrusor overactivity, such as sensations of urinary urgency, pain, and frequency, have common pathological inputs, including high-level sensory nerve signals induced by tissue damage, nerve hypersensitivity, and inflammation [18, 19]. In addition, small myelinated A-delta and unmyelinated C fibers are involved in these symptoms. Some studies have indicated that detrusor overactivity is mediated by nerve hypersensitivity associated with functionalized afferent C fibers [20, 21]. It has also been reported that sensory signals from the bladder under pathological storage conditions associated with inflammation are mediated by afferent C fibers, whereas afferent A-delta fibers mediate normal signaling [22]. Controlling sensory nerve hypersensitivity associated with afferent A-delta and C fibers is important for managing symptoms associated with cystitis.

Thus, we hypothesized that PBM might improve detrusor overactivity and the urgency caused by detrusor overactivity associated with cystitis by controlling sensory nerve hypersensitivity. In addition, laser irradiation selectively decreased the peripheral nerve activity associated with afferent A-delta and C fibers [13–17]. These findings indicate the potential of PBM to control sensory nerve hypersensitivity without influencing efferent nerve activity.

We aimed to study the effects of PBM on the urinary storage dysfunction and voiding function associated with cystitis. Intravesical administration of acetic acid in rats induces a decrease in the voiding interval and bladder capacity during urine storage [20, 23]. Several studies have indicated that the urinary symptoms of acetic acid-induced cystitis in rats are mediated by nerve hypersensitivity associated with afferent A-delta and C fibers [21, 24]. We examined the effects of laser irradiation of the L6 lumbosacral nerve, which contains A-delta and C fibers that transmit the sensation of the bladder [25–27], on micturition reflex parameters in a rat model of cystitis induced by an intravesical infusion of acetic acid.

## Materials and methods

### Animals

Eight-week-old Sprague-Dawley (SD) male rats (Crl:CD, Charles River Co., Ltd.) were used in all experiments. The rats were kept under environmental control with a 12 h light/dark cycle (lights on at 7:00 a.m.), a temperature (permissive range) of 20–26°C, and a humidity (permissive range) of 35–60% with free access to food and water.

### Preparation of animal model

**Fig 1:**
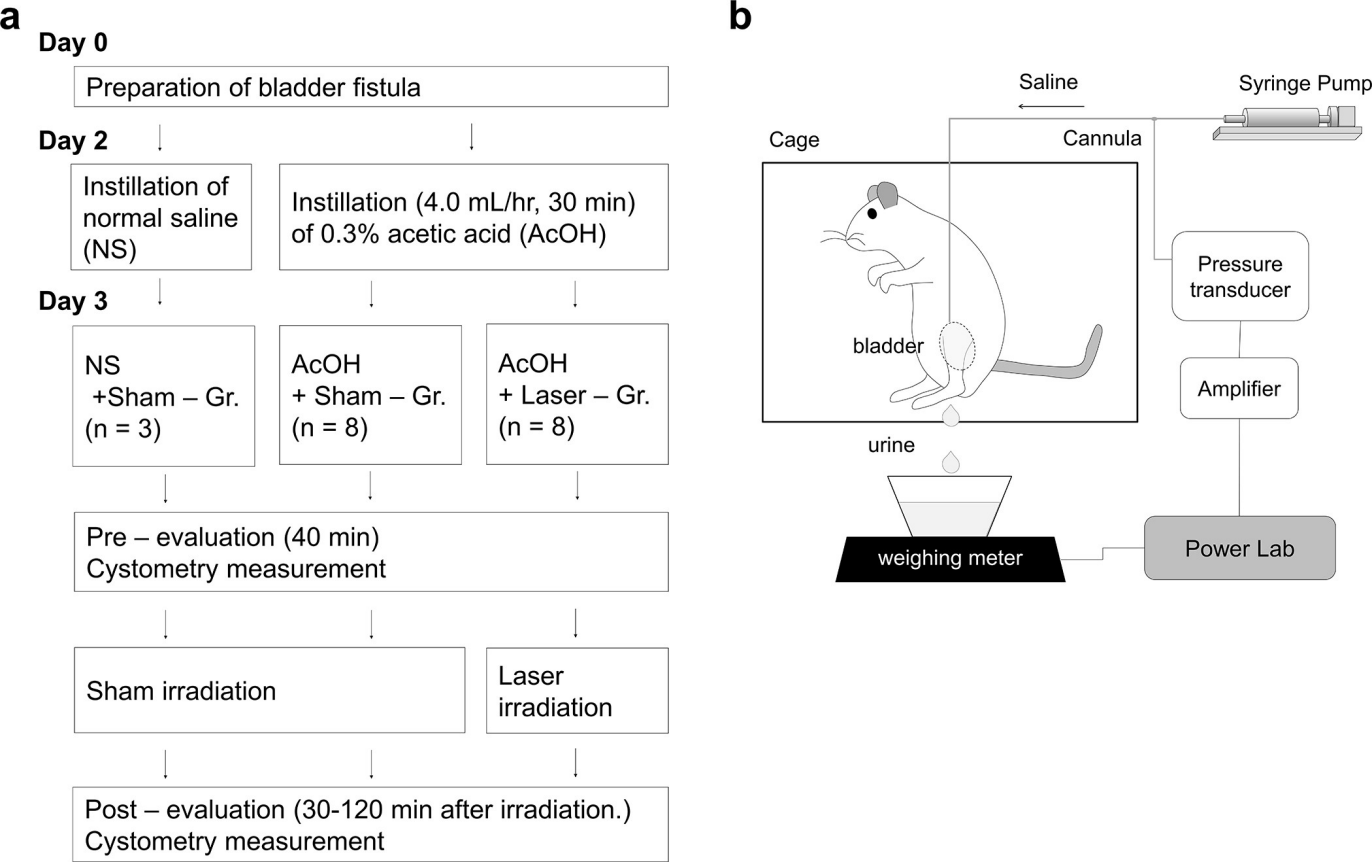
(a) Time course of our experiment. (b) Schematics of our cystometry measurement system. Voiding urine volume and bladder pressure were measured simultaneously with continuous saline infusion through cannula. These measurements were performed under free moving.

The time-course of the experiment is shown in Fig 1A. The rats were anesthetized by inhalation of 2% isoflurane and maintained in the dorsal position. The bladder was exposed using a ventral incision, and a cannula (PE-50; Becton, Dickinson & Co.) was connected to the bladder dome. The other end of the cannula was passed backwards subcutaneously and sutured to the muscles and skin. Outside the body, the cannula was protected using a metal spring and connected to a siebel. Vital signs were monitored frequently from the onset of anesthesia until the rats regained consciousness. Buprenorphine 0.01 mg/kg (Ohtsuka Pharma Ltd.) was subcutaneously administered before and after surgery for pain relief. Two days after surgery, 0.3% acetic acid (AcOH, Wako Pure Chemical Corporation) in saline was injected into the bladder through the cannula and connected to the bladder for 30 min at an infusion rate of 4.0 mL/h. Alternatively, saline was injected into the normal saline with sham irradiation (NS + Sham) group using the same protocol.

### Continuous cystometry

Schematics of our cystometry measurement system are shown in Fig 1B. Three days after surgery, bladder pressure was measured continuously using a transducer with an amplifier (ML117, ADInstruments, Ltd.) and recorded using an A/D converter (PowerLab system, ADInstruments, Ltd.) by transducing the cannula connected to the bladder. Voiding urine volume was measured continuously using a weighing meter under the cage. These measurements were performed with the animal freely mobile and simultaneously with continuous saline infusion at 3.0 mL/h and a constant speed through a T-shaped stopcock connected to the cannula.

Three groups were established—the NS+Sham group (saline infusion and sham irradiation, *n* = 3), the AcOH with sham irradiation (AcOH+Sham) group (AcOH infusion and sham irradiation, *n* = 8), and AcOH with laser irradiation (AcOH+Laser) group (AcOH infusion and laser irradiation, *n* = 8). To evaluate the effect of the laser with an appropriate evaluation range between the NS+Sham and AcOH groups, rats that had voiding intervals less than 10 min before laser irradiation were selected and enrolled uniformly in the AcOH+Sham group and AcOH+Laser group. Among a total of 38 animals, 19 were designated for evaluation and the remaining 19 were held in reserve. Exclusion criteria led to the removal of 15 rats from the evaluation group: 10 were excluded due to voiding intervals exceeding the standard 10-min threshold, 4 were excluded owing to tube breakage during cystometry procedures, and 1 was excluded due to overflow incontinence that precluded evaluation during cystometry measurements. 4 rats not enrolled in any group were euthanized under 2% isoflurane anesthesia. Cystometry was performed continuously for 60 min before and 120 min after laser irradiation. Residual urine was extracted from all the rats enrolled in the groups after cystometric measurement.

### Laser irradiation

The laser conditions were set based on a previous report [28]. An 830 nm laser diode (Sheep, Unitac Inc.) was used as the light source. The irradiation parameter settings were 1 W average output, 10 W peak output, 180 s duration per each side (total: 360 s), 180 J energy per each side (total: 360 J), 1.5 cm^2^ beam area, 667 mW/cm^2^ average power density, 120 J/cm^2^ energy density per each side (total: 240 J/cm^2^), 10% pulse duty cycle, 20 ms pulse width, and 5 Hz frequency. Laser irradiation was applied to the skin on both sides of the L6 lumbosacral intervertebral foramen. The rats in the AcOH+Laser group were kept awake and retained during laser irradiation. Rats in the NS+Sham and AcOH + Sham groups were otherwise identically managed to the rats in the AcOH+Laser group, except for laser irradiation. The hair around the irradiated area of the skin was removed before laser irradiation in all groups.

### Data analysis

Voiding intervals, urine volume, basal bladder pressure, and voiding bladder pressure were analyzed using a cystometry chart for each voiding, and the mean value was calculated for each rat, both before and after laser irradiation. Only data from 40 min before laser irradiation and from 30 to 120 min after laser irradiation were used to remove noise on the bladder pressure chart induced by intense body motion associated with the stress of retaining and holding the rat.

Prism v8.4.3 (Graph Pad Software Incorporated, San Diego, CA, USA) was used for the statistical analysis. Data are presented as the mean +/- standard error of the mean (S.E). For statistical analyses, we performed a one-way analysis of variance (ANOVA) followed by Bonferroni’s post-hoc comparisons tests or a two-way ANOVA followed by Bonferroni’s post-hoc comparisons tests; *p* < 0.05 was considered significant.

## Results

**Fig 2:**
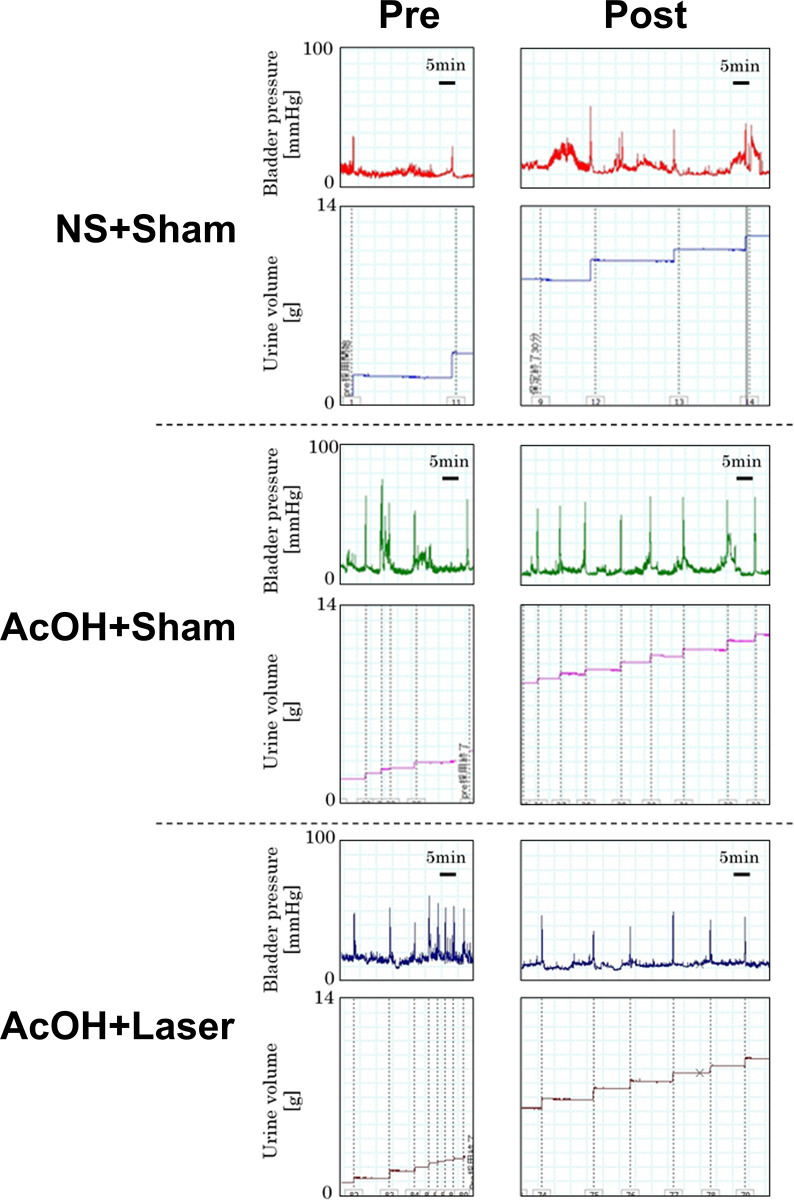
Typical examples of cystometry charts on NS+Sham, AcOH+Sham, and AcOH+Laser group. Pre: Charts before laser irradiation, Post: Charts after laser irradiation; NS, normal saline; AcOH, acetic acid.

**Fig 3:**
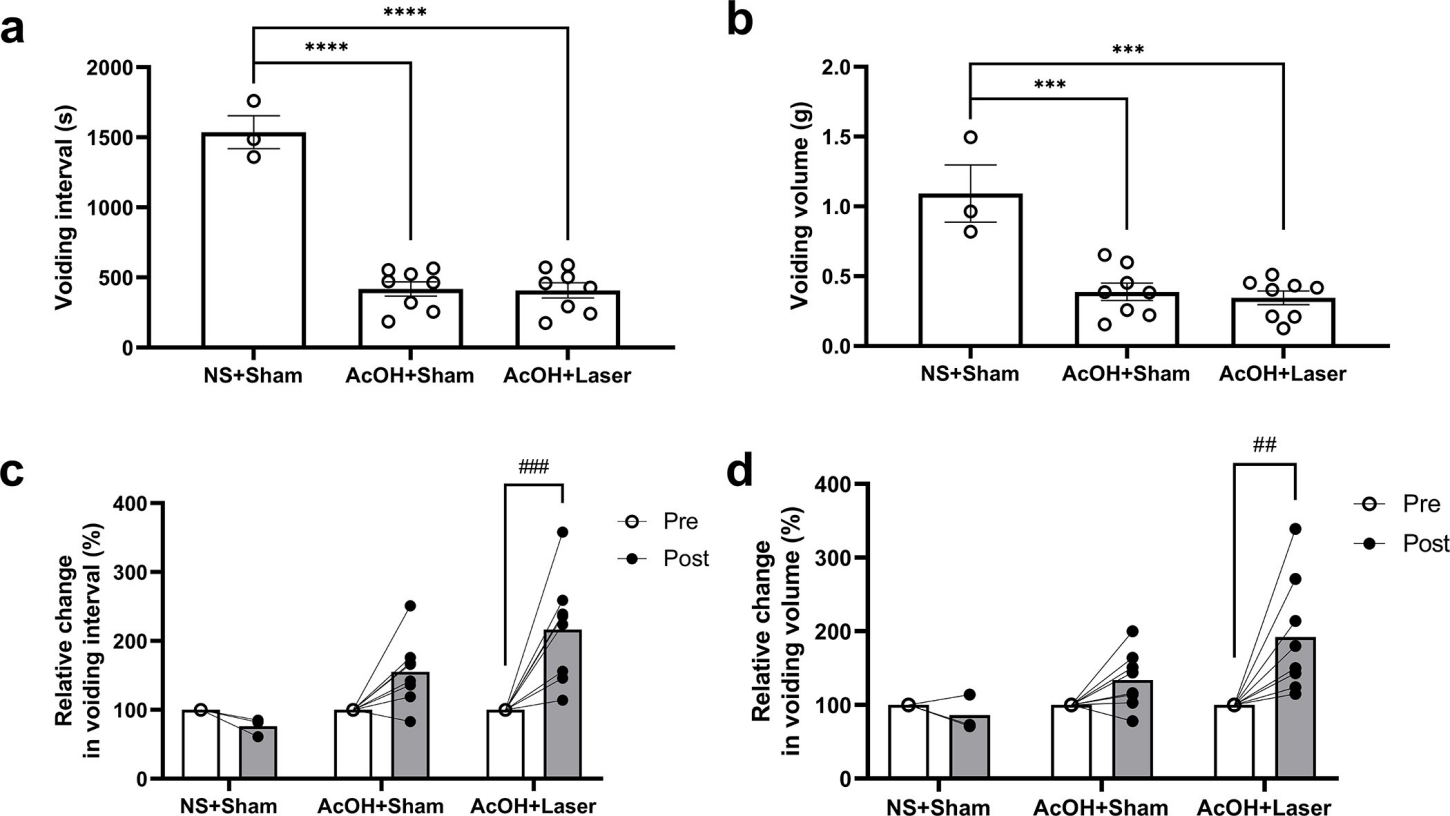
Effect of laser irradiation on micturition reflection parameters in rats. (a, b) Prior to sham or laser irradiation (Pre), the voiding interval and voiding volume significantly decreased in the AcOH+Sham and AcOH+Laser groups, which received a 0.3% acetic acid instillation, compared to the NS+Sham group. There was no significant difference between the AcOH+Sham and AcOH+Laser groups. (c, d) Changes after sham or laser irradiation (post), expressed as a percentage relative to the pre-values. There were no significant changes in the voiding interval and voiding volume in the NS+Sham and AcOH+Sham groups between pre and post. The AcOH+Laser group showed a significant increase in both voiding interval and voiding volume. Pre: Values before sham or laser irradiation, Post: Values after sham or laser irradiation. Values represent mean +/- S.E (*n* = 3 or 8); individual data points are shown; ***: *p* < 0.001 and ****: *p* < 0.0001 vs. NS+Sham group using one-way ANOVA, followed by Bonferroni’s multiple comparisons tests; ##: *p* < 0.01 and ###: *p* < 0.001 vs. Pre using two-way ANOVA followed by Bonferroni’s multiple comparisons tests; NS, normal saline; AcOH, acetic acid.

Typical cystometry charts for the NS+Sham, AcOH+Sham and AcOH+Laser groups are shown in Fig 2, and the voiding interval and volume for each group are shown in Fig 3. Prior to sham or laser irradiation (pre), the voiding interval significantly decreased in the AcOH+Sham (417.9 +/- 51.2 s, *p* < 0.0001) and AcOH+Laser (408.0 +/- 54.3 s, *p* < 0.0001) groups, which received a 0.3% AcOH injection, compared to the NS+Sham group (1536.4 +/- 117.9 s) (Fig 3A). The voiding volume also significantly decreased in the AcOH+Sham (0.388 +/- 0.063 g, *p* = 0.0002) and AcOH+Laser (0.345 +/- 0.050 g, *p* = 0.0001) groups, compared to the NS+Sham group (1.093 +/- 0.205 g) (Fig 3B). There was no significant difference between the AcOH+Sham and AcOH+Laser groups in both of the voiding interval and voiding volumes (Fig 3A and 3B). The reduction in the voiding interval and volume due to AcOH indicated that the symptoms of cystitis were induced.

To evaluate the effects of sham or laser irradiation, the values before irradiation (pre) were set to 100%, and the values after irradiation (post) were calculated. The values before and after sham or laser irradiation were compared. There were no significant changes in the voiding intervals in the NS+Sham (76%) and AcOH+Sham (155%) groups between pre and post. The AcOH+Laser group showed a significant increase in voiding interval between pre- and post (217%; *p* = 0.0002). There were also no significant changes in the voiding volume in the NS+Sham (86%) and AcOH+Sham (134%) groups between pre and post. The AcOH+Laser group showed a significant increase in voiding volume between pre and post (192%; *p* = 0.0012).

**Fig 4:**
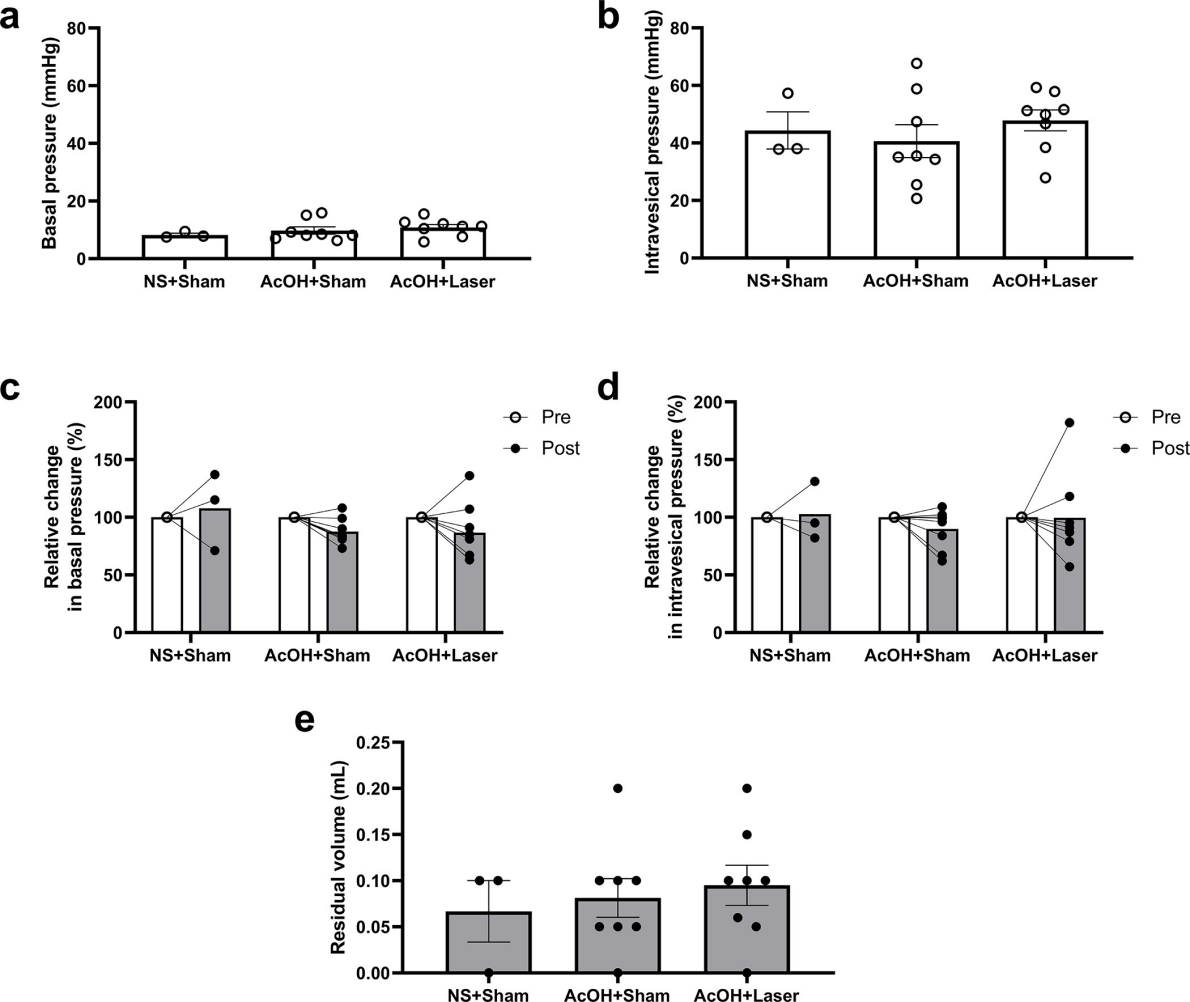
(a, b) Prior to sham or laser irradiation (Pre), there were no significant differences in basal pressure and intravesical pressure among the NS+Sham, AcOH+Sham, and AcOH+Laser groups. (c, d) Changes after sham or laser irradiation (Post), expressed as a percentage relative to the pre-values. There were no significant changes in any of the groups between Pre and Post. (e) Residual urine volume. There were no significant differences among any of the groups. Pre: Values before sham or laser irradiation, Post: Values after sham or laser irradiation. Values represent mean +/- S.E (*n* = 3 or 8); individual data points are shown; one-way ANOVA followed by Bonferroni’s multiple comparisons tests (a, b, e) or two-way ANOVA followed by Bonferroni’s multiple comparisons tests (c, d) were used; NS, normal saline; AcOH, acetic acid.

To evaluate the effects of the laser on voiding function, basal pressure, intravesical pressure, and residual urine volume were evaluated (Fig 4). At pre irradiation, no difference in basal pressure was observed among the NS+Sham (8.2 +/- 0.6 mmHg), AcOH+Sham (9.8 +/- 1.3 mmHg), and AcOH+Laser groups (10.8 +/- 1.1 mmHg) (Fig 4B). There was also no difference in intravesical pressure among the NS+Sham (44.4 +/- 6.5 mmHg), AcOH+Sham (40.6 +/- 5.7 mmHg), and AcOH+Laser groups (47.9 +/- 3.7 mmHg) (Fig 4B). Between the pre- and post-irradiation, there were no significant changes in basal pressure among the NS+Sham (108%), AcOH+Sham (88%), and AcOH+Laser groups (87%) (Fig 4C). There were also no significant changes in intravesical pressure among the NS+Sham (103%), AcOH+Sham (90%), and AcOH+Laser groups (100%) (Fig 4D). Residual urine volumes in the NS+Sham, AcOH+Sham, and AcOH+Laser group were 0.07 +/- 0.03 mL, 0.08 +/- 0.02 mL, and 0.10 +/- 0.02 mL, respectively (Fig 4E).

## Discussion

Our study showed that urinary storage dysfunction was improved by laser irradiation without impairing the voiding function. Ito et al. reported that 830 nm laser diode irradiation suppressed bladder hyperactivity associated with the hypersensitivity of afferent nerves in a rat cystitis model induced by AcOH and PGE2 [28]. In their study, in rats with cystitis restrained in Bollman cages, the voiding interval measured by cystometry was prolonged after laser irradiation, corresponding to diminished bladder hyperactivity. These findings were consistent with our results. In our study, we compared three groups, the NS+Sham group (fistula preparation and saline infusion), AcOH+Sham group (fistula preparation and AcOH infusion), and AcOH+Laser group (fistula preparation, AcOH infusion, and laser irradiation), to remove any bias related to a change in the voiding interval due to long-term cystometry. In addition, cystometry was conducted with the animals freely mobile to remove any effects that might shorten the voiding interval due to restraint stress of restraint [29]. These methodologies contribute to increasing the reliability of experiments. Moreover, we observed an increase in voiding volume in the AcOH+Laser group, thereby elucidating the role of the laser in enhancing urinary storage dysfunction. Furthermore, there were no significant changes in basal or intravesical pressure following laser irradiation, and no alteration in residual urine volume occurred, indicating that voiding function was preserved. These findings indicate that PBM is an effective and safe treatment option for patients with cystitis.

In addition, laser irradiation did not affect bladder contraction during the voiding phase, despite improving bladder hyperactivity during the storage phase. In Ito’s study [28], laser irradiation did not alter the maximum intravesical pressure, although it prolonged voiding intervals in a rat cystitis model. This finding also supports our results, implying that laser irradiation may improve urine storage without the loss of voiding function. We consider that the selective control of the storage dysfunction resulting from the laser effect of 830 nm laser diode irradiation was due to the selective inhibition of afferent C and A delta fibers, but not efferent B fibers. The L6 lumbosacral nerve that projects to the bladder contains various nerve fibers, among which C and A-delta fibers transmit the sensation of the bladder to the center during the urinary storage period and efferent B fibers control bladder contraction during the voiding phase [25–27]. In a study by Ito et al., electrophysiological measurements of the L6 dorsal root ganglion activity showed that sensory nerve activity was suppressed by laser [28]. According to other electrophysiological studies, Tsuchiya et al. reported that 830 nm laser irradiation diminished the slow component of axonal volleys to dorsal roots from the saphenous nerve in rats, but did not affect the fast component of axial fibers [13]. They also reported that 830 nm laser irradiation depressed neuronal responses to nociceptive stimuli but did not alter the responses elicited by brush stimulation [14]. Other previous studies, which examined the effects of lasers on the firing frequency in the rat spinal dorsal horns evoked by mechanical stimulation, suggested that lasers did not affect the activity of A-beta fibers, but selectively inhibit the activity of C and A-delta fibers [15–17]. These results indicate that 830 nm laser irradiation selectively suppresses afferent C and A-delta fiber activity in our rat cystitis model. Selective inhibition of afferent C fand A-delta fibers by laser normalizes AcOH-induced hyperactivation of the micturition reflex, which may improve storage dysfunction. In contrast, bladder contraction during the voiding phase is controlled by efferent B-fibers, which have a relatively high conduction velocity [26]. Laser irradiation did not change the basal or intravesical pressure (Fig 4C and 4D), suggesting that the laser did not affect the activity of efferent B fibers. This may explain why laser irradiation did not affect bladder contraction during the voiding phase despite improving bladder hyperactivity during the storage phase.

The 830-nm laser was used to percutaneously irradiate the L6 lumbosacral intervertebral foramen at a power density of 667 mW/cm^2^ and an energy density of 120 J/cm^2^. In our previous report [30], the light penetration of the 830 nm laser used in this study was determined in the lumbosacral nerves of rats. We obtained a correlation between the depth of the nerves from the skin surface and the power density measured by implanting photodiodes near the lumbosacral nerves in rats. Laser power density and fluence at lumbosacral nerves were calculated as 23.2 mW/cm^2^ and 4.2 J/cm^2^ for these experimental conditions, respectively. According to the review by Huang, PBM effects appear to be obtained in the dose range of around 0.01–10 J/cm^2^ [2]. These findings confirm that a sufficient laser dose reached the lumbosacral nerves.

This study had several limitations. First, the mechanism underlying the selective inhibitory effect of PBM on nerve fibers remains unknown. This study suggests that PBM may have selectively suppressed A-delta and C fibers in a rat model of cystitis induced by AcOH. Future studies focusing on molecules specific to A-delta and C fibers may allow us to investigate the molecular mechanisms of PBM. Second, the dependence of the laser conditions on efficacy is unknown. A previous study reported that 808 nm PBM of L6 dorsal root ganglion with average power of 18–460 mW and energy of 10.8–276 J improved visceral hyperalgesia induced by restraint stress [31]. Although the animal model used in this study and the previous study differ, it is possible that similar mechanisms of action of PBM may have occurred. Therefore, it is likely that there are more effective treatment conditions for the cystitis model as well. Wavelength, power, and energy may have optimal therapeutic effects. Third, although a single irradiation was performed in this study, the persistence of the effect over 2 h is unknown, as is the accumulation of the effects of multiple irradiations. In pain treatment, the accumulation and persistence of effects are observed with multiple laser irradiations [7]. Assuming that the treatment mechanism suppresses the neural activity of A-delta and C-fibers as in the case of pain treatment, the effect may accumulate in the case of urinary symptoms as in the case of pain treatment. In the evaluation using cystitis model rats, the cystometry method used in this study is difficult to evaluate over multiple days. It may be possible to evaluate the effects of multiple irradiations using an evaluation method that allows spontaneous urination to be performed without creating a bladder fistula or saline infusion.

## Conclusions

In this study, we investigated the effect of PBM on storage dysfunction and voiding function in rats with AcOH-induced cystitis subjected to transcutaneous laser irradiation of the lumbosacral nerves. Laser irradiation resulted in a significant increase in voiding interval and voiding volume, suggesting that bladder hyperactivity during the storage phase was improved by laser irradiation. Laser irradiation did not affect bladder contraction during the voiding phase. These results indicate that laser irradiation improves urinary storage dysfunction without the loss of voiding function. PBM may improve lower urinary tract dysfunction associated with detrusor overactivity.

## Supporting information

S1 Data (XLSX)
